# Phase I/II clinical trial using HLA-A24-restricted peptide vaccine derived from KIF20A for patients with advanced pancreatic cancer

**DOI:** 10.1186/1479-5876-11-291

**Published:** 2013-11-16

**Authors:** Shingo Asahara, Kazuyoshi Takeda, Kenji Yamao, Hiroyuki Maguchi, Hiroki Yamaue

**Affiliations:** 1 Department of Internal Medicine, Chiba Tokushukai Hospital, Chiba, Japan; 2 Department of Immunology, Juntendo University School of Medicine, Tokyo, Japan; 3 Department of Gastroenterology, Aichi Cancer Center Hospital, Nagoya, Japan; 4 Center for Gastroenterology, Teine-Keijinkai Hospital, Sapporo, Japan; 5 Second Department of Surgery, Wakayama Medical University School of Medicine, Wakayama, Japan

**Keywords:** KIF20A, Peptide vaccine, Pancreatic cancer

## Abstract

**Background:**

We previously developed an immunotherapy treatment utilizing a cancer vaccine reagent KIF20A-66 in order to treat pancreatic cancer. KIF20A-66 is HLA-A24-restricted epitope peptide derived from KIF20A, a member of kinesin super family protein 20A that is significantly transactivated in pancreatic cancer. In this report, we further demonstrated non-randomized, open-label, single centered phase I/II clinical trial of immunotherapy using the KIF20A-66 peptide for the patients with advanced pancreatic cancer.

**Methods:**

Vaccination was performed to the patients with metastatic pancreatic cancer, in whom gemcitabine-based therapy had failed. In phase I study, KIF20A-66 peptide was subcutaneously injected weekly in a dose-escalation manner (doses of 1.0 and 3.0 mg/body, 6 patients/1 cohort). After safety was assessed, phase II study was conducted using 3.0 mg of KIF20A-66 peptide.

**Results:**

KIF20A-66 peptide vaccination was well tolerated in the doses we examined and tumor responses after 1 month of the treatment were evaluated. Among 29 patients who completed one course of the treatment at least, stable disease (SD) was found in 21 cases, while progressive disease (PD) was found in 8 cases, indicating that the disease control rate was 72%. Objective tumor shrinkage was observed in 8 cases, including 1 case of complete response (CR). The median survival time (MST) and progression free survival time (PFS) were 142 days and 56 days, respectively. These results clearly demonstrate that overall survival of the patients was significantly prolonged, compared to the historical controls of 9 cases with unmatched HLA in the same hospital (MST: 83 days), as well as 81 cases in our and other hospitals (MST: 63 days).

**Conclusion:**

The patients vaccinated with KIF20A-66 peptide had better prognosis than the control group with best supportive care (BSC). Thus, we concluded that KIF20A-66 vaccination is significantly effective as an immunotherapy against advanced pancreatic cancer. KIF20A-66 peptide was well tolerable in the dose of either 1.0 mg or 3.0 mg/body, and effectively induced peptide-specific response of cytotoxic T lymphocyte (CTL). Further clinical study using this peptide is a promising approach for advanced pancreatic cancer to achieve high potential benefit for better prognosis.

**Clinical trial registration:**

UMIN-CTR, number UMIN000004919

## Introduction

Pancreatic cancer remains one of the most challenging conditions to treat, due to extremely poor prognosis with the overall five-year survival of less than 10% [1-3]. During the last decades, gemcitabine has been the standard single-agent chemotherapy for unresectable pancreatic cancer [4,5]. Regarding combination chemotherapy, several phase III trials of gemcitabine-based multi-drug regimens have been attempted, whereas significant improvement in survival has not been observed [6-14]. Although TS-1, a prodrug of 5-FU, has been employed as a major alternative approach in a variety of solid tumors, the single-agent treatment of TS-1 yielded non-inferiority result against the gemcitabine treatment [15]. After all, once pancreatic cancer became refractory to gemcitabine, there is virtually no effective treatment for the patients. Hence, novel strategy providing better survival benefit is urgently required, in particular, for the patients with advanced pancreatic cancer.

Cancer immunotherapy is a promising approach to fight against cancer, and thus we have conducted research and development of peptide vaccines targeting tumor-specific antigens [16-19]. Briefly, we identified dozens of cancer-testis or oncofetal proteins from more than 1,000 clinical cancer tissues using cDNA microarray including 32,000 genes or ESTs [20]. Utilizing the result of this genome-wide expression profile analysis, we tried to establish an epitope peptide derived from the tumor-associated antigen mentioned above, which is applicable for cancer peptide vaccination [21,22]. KIF20A, kinesin family member 20A, is one of the candidates of such target antigen, as it was up-regulated in the majority of pancreatic cancer [23]. Therefore, we developed an epitope peptide, namely KIF20A-66, restricted to HLA-A*2402 that is the most common HLA-A allele in a Japanese population [24]. We here report the results of a phase I/II clinical trial using KIF20A-66 mono peptide as cancer immunotherapy for the patients with advanced pancreatic cancer.

## Methods

### Patient eligibility

Patients with unresectable or metastatic pancreatic cancer, who were resistant to gemcitabine and TS-1 treatments or unable to continue the treatment of gemcitabine or TS-1 because of severe adverse events, were enrolled in this trial from March 2009 to February 2010 at Chiba Tokushukai Hospital. The eligibility criteria are as follows: unresectable pancreatic cancer with metastatic, recurrent and/or locally advanced disease based on diagnostic imaging using computed tomography (CT) and histological examinations. Other entry criteria included the HLA-A*2402-positive status, an Eastern Cooperative Oncology Group (ECOG) performance status of 0–2, age of 20–85 years, life expectancy of at least 2 months, adequate respiratory, and liver and kidney functions for vaccination treatment. The exclusion criteria are as follows: pregnancy or lactation, active infection, other active malignancy, non-recovered injury, and treatment with immunosuppressive agents or steroid. Written informed consent was obtained from each individual patient, and the study was approved by Tokushukai Group Ethical Committee. The study was registered at University Hospital Medical Information Network (UMIN) Center with the Clinical Trial Registration number UMIN000004919.

### Control group

Clinical data used as the control group (BSC, multi-center, n = 81) in this study were obtained from our and other hospitals where written informed consent was obtained at each institution. Clinical information of each patient utilized in our statistical analysis includes age at diagnosis, sex, performance status at the endpoint of the Standard Chemotherapy, treatment status at primary lesion, median survival time, and mean survival time. This study was approved by the institutional review board at each institution.

### Study design and end points

This study is a non-randomized, open-label phase I/II clinical trial with dose escalation of KIF20A-66 peptide mono-therapy. The primary end point of phase I part was safety of peptide vaccination and tolerance for phase II part. The primary end point of phase II part was antitumor effects assessed by CT scan in accordance with the Response Evaluation Criteria in Solid Tumors (RECIST) criteria version 1.1. The secondary end points were overall survival (OS), progression free survival (PFS), immunological responses assessed by CTL induction specific to the KIF20A-66 peptide and the injection site reactions (ISRs). In phase II part, the information of 9 patients with best supportive care in the Chiba Tokushukai Hospital from January 2007 to January 2009 was used as a historical control.

### Treatment protocol

After emulsified with Incomplete Freund’s adjuvant (Montanide ISA51VG, SEPPIC, France), KIF20A-66 peptide in the amount of 1.0 or 3.0 mg/body was subcutaneously administered on days 1, 8, 15 and 22 in a 28 days-treatment cycle. After two cycles of the vaccination, the peptide was administrated once in every two weeks until tumor progression was observed in the patient.

### Toxicity assessment

The toxicity was assessed based on the Common Terminology Criteria for Adverse Events version 3.0 (CTCAE v3.0).

### Peptides

The KIF20A-66 peptide (KVYLRVRPLL) was synthesized and its quality was analyzed by American Peptide Company Inc. (Sunnyvale, CA). The epitope peptide derived from HIV-Env peptide (RYLRDQQLL), restricted to HLA-A*2402, was used as a control to evaluate CTL response.

### Enzyme-linked immunospot (ELISPOT) assay

To evaluate the peptide-specific CTL response, ELISPOT assay was performed after *in vitro* sensitization [16]. Briefly, frozen Peripheral Blood Mononuclear Cells (PBMC) derived from the same patient were thawed, cultured with respective peptide and IL-2 (Novartis, Emeryville, CA) (IVS), and harvested after two weeks. Followed by CD4^+^ cell depletion, IFN-γ ELISPOT assay was performed utilizing HLA-A*2402-positive TISI cells (IHWG Cell and Gene Bank, Seattle, WA) stimulated by either vaccinated peptide or HIV-Env peptide (as control). Reaction in a MultiScreen-IP 96-plate (Millipore, Bedford, MA) was measured by an automated ELISPOT reader, ImmunoSPOT S4 (Cellular Technology Ltd, Cleveland, OH) with ImmunoSpot Professional Software Version 5.0 (Cellular Technology Ltd). All ELISPOT assays were performed in triplicate. The number of peptide-specific spots was calculated by subtracting the number of the spots of control cells from that of the cells stimulated by vaccinated peptide. The peptide-specific T cell response was classified into four grades (−, +, ++, and +++), according to the algorithm flow chart described in our previous report (+++ : the content rate of CTL is more than 0.2% , ++ : 0.02 - 0.2%, + : 0.01 - 0.02%, –: less than 0.01%) [25]. Sensitivity of ELISPOT assay was estimated as approximate average level utilizing proficiency panels conducted by Cancer Immunotherapy Consortium (CIC) in 2009 and 2011 [26].

### Flow cytometry

Expression of peptide specific T cell receptor (TCR) was examined by FACS-CantoII (Becton Dickinson, San Jose, CA) using KIF20A-66/HLA-A*2402 dextramer-PE (KIF20A-dextramer) according to the manufacturer’s instruction (Immudex, Copenhagen, Denmark). HIV-A24 epitope peptide (RYLRDQQLL)/MHC-dextramer (HIV-dextramer) was used as negative control. Briefly, cells were incubated with peptide-HLA-A*2402 dextramer-PE for 10 minutes at room temperature, then treated with FITC-conjugated anti-human CD8 monoclonal antibody (mAb), APC-conjugated anti-human CD3 mAb, PE-Cy7-conjugated anti-human CD4 mAb, and 7-AAD (BD Biosciences, San Jose, CA) at 4°C for 20 minutes. Analysis gate was set on the staining profiles using HIV-dextramer, and positive cell percentage (dextramer^+^ cells/CD3^+^ CD4^-^ CD8^+^ cells) was calculated by subtracting the percentage of HIV-dextramer^+^ from that of KIF20A-dextramer^+^.

### Statistical analysis

StatView version 5.0 (SAS Institute Japan Ltd., Japan) was used for statistical analysis. TTP and OS curves were estimated using the Kaplan-Meier methodology and analyzed with a log-rank test. Mann–Whitney U test and Chi-square test were used to compare patient characteristics.

## Results

### The peptide vaccine treatment

A total of 31 patients with chemotherapy-refractory pancreatic cancer were enrolled in this trial. 16 patients had unresectable tumor and 15 had recurrent one after surgery. Tables 1 and 2 indicate clinicopathological information of the 31 patients, as well as the patients in control group, who received best supportive care in our and other hospitals (Table 1). The peptide in the amount of either 1.0 mg or 3.0 mg per body was examined in this phase I/II study. These dosages were well tolerated in the 31 patients with advanced pancreatic cancer. There is no severe adverse event (SAE) related to the peptide vaccine in the 1.0 mg/body-injected group, except the immunological response at injection sites. As well, no SAE was observed in the first 6 patients in the 3.0 mg/body-injected group during the first cycle in the treatment. Hence, we determined that 3.0 mg per body is an appropriate dose for phase II part in this study.

**Table 1:** Clinical status and profile of the patients

	**KIF20A peptide vaccine treatment**	**Best supportive care**	
	Chiba (n = 31) *	Chiba (n = 9) *	Multi-center (n = 81) **
Age (average, (range))	61.3 (33–80)	64 (53–82)	64.5 (41–85)
Sex (Male: Female)	17:14	5:4	49:32
Performance status (0:1:2:3)	11:8:12:0	1:3:3:2	13:28:36:0 ***
Status of primary lesion (Resected: Unresected)	15:16	1:8	23:58
Median survival time (days)	142.0 ± 23.7	83.0 ± 33.5	62.0 ± 6.5
Mean survival time (days)	171.8 ± 23.8	93.3 ± 14.8	91.1 ± 11.6

*, Clinical data obtained at our institution, Chiba Tokushukai Hospital.

**, Clinical data of Multi-center (n = 81) include those obtained from Chiba and other three hospitals.

***, 4 cases were excluded, since Performance Status was not determined.

**Table 2:** Patient characteristics and clinical responses

**No.**	**Age**	**Sex**	**Target lesion**	**Dose of peptide (mg)**	**Number of injection**	Clinical response*	**Objective Response**	**Response lesion**	**Injection site reaction(Grade)**	**CTL response**		**PFS(day)**	**OS (day)**	**Pre-vaccination**			**Post-vaccination**		
										**Pre-vaccination**	Post-vaccination**			**WBC(/mm**^**3**^**)**	**Lymphocyte (%)**	**Lymphocyte (/mm**^**3**^**)**	**WBC(/mm**^**3**^**)**	**Lymphocyte (%)**	**Lymphocyte (/mm**^**3**^**)**
1	75	M	Local LNs	1	4	PD			0	N.A.	+	36	36	7300	7	511	5300	10.5	557
2	57	F	Local	1	11	PD			1	++	++	26	108	7400	13	962	7900	8	632
3	72	M	Liver	1	3	-			0	N.T.	N.T.	31	31	7800	11	858	16200	10.5	1701
4	60	M	Lung, local LNs	1	19	SD	Yes	Lung metastasis	2	+	-	223	283	5100	21	1071	5200	10.5	546
5	72	F	Primary , liver	1	12	PD			1	+	+++	24	128	2400	25.5	612	4200	10.8	454
6	65	F	Liver	1	4	PD			0	+	+	26	40	4500	16.5	743	8000	4.1	328
7	61	F	Local , liver	3	14	SD			2	+	+++	55	155	4000	25	1000	6400	18.3	1171
8	57	F	Primary, liver	3	10	SD			2	++	+++	56	145	4500	33	1485	14100	16	2256
9	33	F	Paraaortic LNs	3	29	SD	Yes(CR)	Liver metastasis	3	N.A.	+++	>1219	>1219	2500	44.5	1113	3600	33	1188
10	76	M	Liver	3	12	PD			2	-	++	27	142	2300	29.5	679	5800	11.5	667
11	55	F	Primary, lung	3	17	SD			1	+	-	112	225	2600	9	234	2200	11.5	253
12	58	M	Primary	3	5	PD			0	-	-	32	32	4500	30	1350	2400	10.7	257
13	58	F	Live, lung, LNs	3	10	SD			1	-	++	57	97	7100	15.5	1101	9100	10.5	956
14	60	M	Liver, LNs	3	17	SD	Yes	Liver metastasis, LNs	2	+++	+++	169	220	2300	27	621	4100	19.5	800
15	80	F	Liver, LNs, lung	3	5	PD			0	-	+	24	44	8500	9.5	808	13300	4.8	638
16	58	M	Primary, liver, lung	3	13	PD			1	-	++	28	182	4800	27	1296	6400	19.3	1235
17	49	M	Parimary	3	17	SD			2	+	+++	169	309	6200	26.5	1643	7900	17.5	1383
18	62	M	Primary, liver, LNs	3	7	SD			1	-	+++	93	93	4200	28	1176	6600	18.6	1228
19	61	M	Primarym, liver, lung, LNs	3	11	SD			2	-	+	57	105	10200	20.5	2091	28700	9	2583
20	58	M	LNs, lung	3	25	SD			2	+	+++	169	332	10100	34	3434	7600	19.5	1482
21	47	M	Primary, liver	3	13	SD			1	-	+	56	249	6000	27.5	1650	9600	8	768
22	71	F	Liver, local LNs	3	7	SD	Yes	Liver metastasis	2	N.A.	++	89	89	7000	11.5	805	5200	20	1040
23	50	M	Local, LNs	3	6	SD			0	N.A.	-	148	148	7900	20	1580	11200	19	2128
24	74	M	Bone	3	21	SD	Yes	Bone metastasis	2	N.A.	+++	415	495	3800	16	608	5600	17.8	997
25	69	F	Primary	3	2	-			0	N.T.	N.T.	11	11	7600	21.5	1634	7400	20.5	1517
26	80	M	Liver, lung	3	18	SD			1	+	+++	112	207	6600	24	1584	7500	21.5	1613
27	44	M	Liver, lung, local LNs	3	24	SD	Yes	Lung and liver metastasis	1	+	-	115	317	2900	23.5	682	4000	25.5	1020
28	61	F	Peritoneal, local LNs	3	9	SD	Yes	Peritoneal metastasis	0	-	-	69	69	9000	26.5	2385	11200	7.5	840
29	46	M	Liver	3	10	SD			2	-	+++	52	388	4000	26	1040	5600	24.6	1378
30	64	F	Liver	3	9	SD			2	-	+++	56	69	4800	26.5	1272	8900	7.1	632
31	68	F	Liver	3	9	SD	Yes	Liver metastasis	2	+	+++	56	82	6800	33	2244	8300	19.5	1619

*Clinical response was evaluated one month after vaccination. PD, Progressive disease; SD, Stable disease; CR, Complete response; OR, Objective response.

**Best CTL response after vaccination. CTL responses were evaluated and classified based on the algorithm as described in Methods.

N.T. (Not Tested); CTL response was not tested in the samples in which PD was observed within one course of the treatment.

N.A. (Not Analyzed); CTL response was not analyzed because of the poor viability during the *in vitro* stimulation.

Immunological injection site reactions (ISRs) of all the 31 patients were evaluated. Clinical responses of 29 patients out of 31, who received at least one treatment cycle (4 injections), were evaluated by immuno-monitoring. ISRs, including adverse reactions on the skin in grades 1–3, was observed in 23 patients out of 29. It should be noted that there were two patients who were incompatible with further vaccination treatment due to the exclusion criteria, such as autoimmune hepatitis and interstitial pneumonia. The patient, who experienced grade 3 autoimmune hepatitis after 11 months of vaccination, was recovered after drug withdrawal. Another patient with the interstitial pneumonia was well recovered by hospital treatment without any steroid therapy. In these cases, we could not rule out the possibility whether these adverse events were related to vaccine treatments or not.

### Clinical outcomes of eligible patients

**Figure 1:**
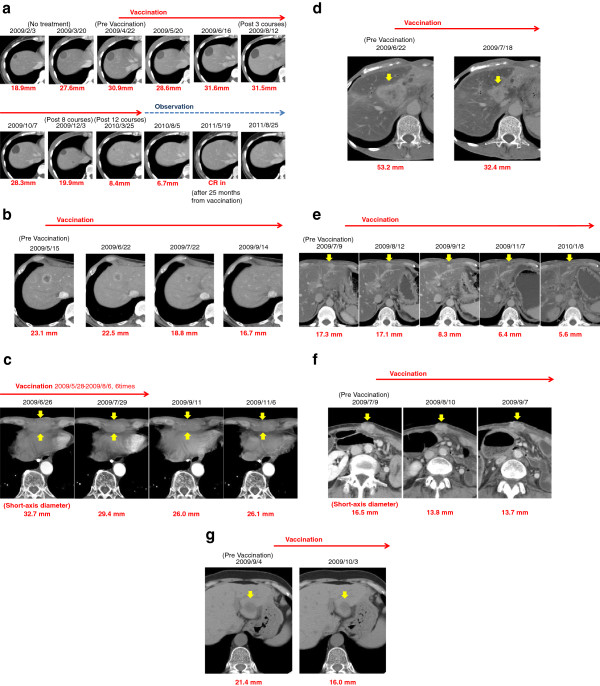
**CT images of the target lesions showing objective tumor response.** Representative CT images of the target lesions were shown, such as liver metastasis in case 9 **(a)**, liver metastasis in case 14 **(b)**, bone metastasis in xiphoid process in case 24 **(c)**, liver metastasis in case 22 **(d)**, liver metastasis in case 27 **(e)**, peritoneal metastasis in case 28 **(f)**, and liver metastasis in case 31 **(g)**. Arrow head indicates the target lesion.

**Figure 2:**
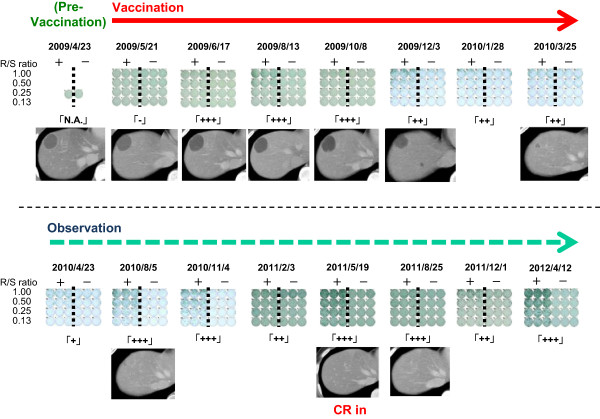
**Peptide specific CTL response in case 9.** Strong CTL responses specific to KIF20A-66 peptide were obtained at the time of 2 months after vaccination. The responses were kept strong positive during 2 years of the observation period. The number of the spots specific to peptide was calculated by subtracting the spot number in control wells from that in peptide-pulsed TISI cells.

**Figure 3:**
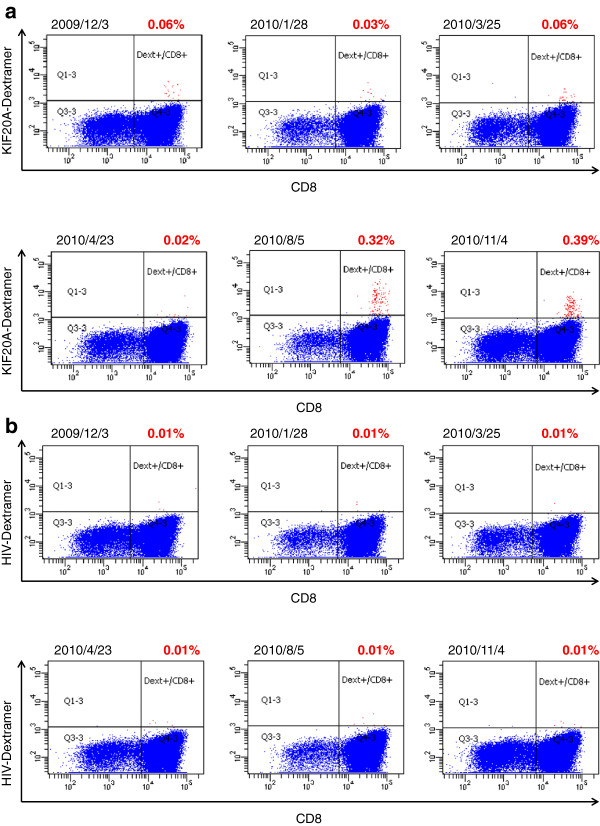
**Flow cytometry analysis of KIF20A-66 specific TCR expression in CD8**^**+ **^**cells in case 9.** Cells were stained with either KIF20A-dextramer **(a)** or HIV-dextramer **(b)** after IVS as described in Methods section. The content rates of KIF20A-dextramer positive or HIV-dextramer positive cells (red dots) in CD3^+^ CD4^-^ CD8^+^ cells are shown above panels in red.

Among the 29 patients examined in this trial, 21 patients yielded the status of “stable disease” (SD), while 8 resulted in “progressive disease” (PD) after one cycle of the treatment (injections of the peptide vaccine for 4 times) (Table 2). The rate of disease control at the time of one cycle was calculated to be 72%. 8 patients showed objective tumor response at target lesions (Figure 1). On the other hand, according to RECIST criteria, the other patients were not classified as partial response (PR), since the ratio of tumor shrinkage was insufficient. One patient (case 9) achieved “complete response” (CR) after SD over the long term (Table 2, Figures 1a, 2, and 3). The rate of objective response to the total was calculated to be 25.8%.

Case 9 describes a 33-year-old female ended up with CR after 25 months including a long period of SD (Figure 1a). This patient underwent pancreatoduodenectomy in November 2008 and was diagnosed with giant cell pancreatic cancer. Adjuvant chemotherapy utilizing gemcitabine was discontinued at the one course of drug administration, due to severe adverse reactions including hematopoietic toxicity. In February 2009, a progressive solitary liver metastasis was diagnosed (Figure 1a). There was no clinical sign of inflammation at the time of April 13th, 2009. White blood cell count (2.8 × 10^3^/µ-l) and CRP level (0.02 mg/dl) were within normal limits. Vaccination started on April 23rd, 2009, and the tumor kept stable condition during the administration. After 8 months, shrinkage of the tumor size was observed. Vaccination was discontinued after 11 months, because the level of liver enzyme was increased and thus autoimmune hepatitis was suspected. Nonetheless, the tumor continued to shrink and became undetectable by CT 25 months after the start of administration. At the time of the submission of this manuscript, there is no sign of relapse or metastasis, and the general condition of the patient has been kept well with the performance status (PS) of zero.

Case 14 reports a 60-year-old male who showed objective response (Figure 1b). After pancreatoduodenectomy, gemcitabine treatment started in October 2008 and liver metastasis was found 3 months later. Followed by TS-1 chemotherapy, we found that metastatic lesions in the liver progressed after the condition of SD during 3 cycles of TS-1 treatment. After 1 cycle of the peptide vaccine, one target lesion of liver metastases located at S8 was shrunken. This lesion kept shrinking until September 2009, and became hardly detectable by CT scan. Similarly, a metastatic lesion in the lymph node was significantly shrunken until September 2009. However, the other target lesion (S4) in the liver showed no response to the vaccine treatment and the tumor progression was promoted after 2 cycles. Finally, the patient died at 220 days after the start of the vaccination.

In case 24, a 74-year-old male also showed objective response (Figure 1c). After distal pancreatectomy in August 2007, adjuvant chemotherapy utilizing gemcitabine was performed for 6 months and then switched to TS-1 because of the side effect. Bone metastasis was found in the xiphoid process by CT scan in April 2009. Radiation therapy was performed to the xiphoid process in May 2009, but the tumor did not respond well. The patient was enrolled into the peptide vaccine trial in July 2009 after one month of cooling off period. Bone metastasis started to shrink after one cycle of the peptide vaccine treatment. The precordial pain was rapidly diminished and well controlled without opioid treatment. After the 5th shot of the peptide, Grade 3 interstitial pneumonia was observed and the treatment was discontinued. The patient was hospitalized in one week of treatment without any steroid therapy and then well recovered. Even without the vaccination, pain was well controlled and tumor markers kept decreasing for the next two months. After the re-progression of the disease, gemcitabine was administered and no clinical effect was observed. Since the patient desired to receive the peptide vaccine again, we obtained an approval of the re-entry of this case from the Ethical committee. The vaccine treatment was restarted with careful monitoring, while neither adverse events nor clinical effect was observed in this second round of drug administration. His overall survival period from the first day of administration was 495 days.

**Figure 4:**
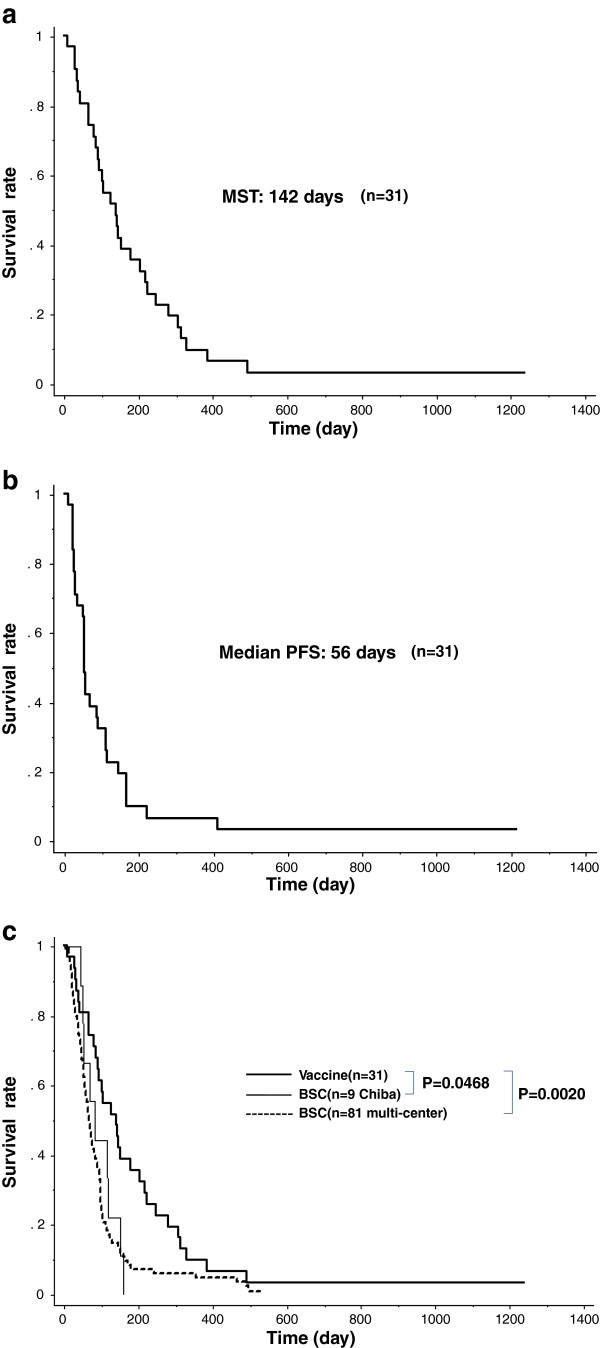
**Overall survival and progression free survival in phase I/II trial.** Overall survival of the patients was shown in Kaplan-Meier plots (n = 31) **(a)**. MST of the patients with peptide vaccine was 142 days. PFS of the patients with peptide vaccine was 56 days **(b)**. In comparison with the control patients who were treated with best supportive care in Chiba Tokushukai Hospital (n = 9), overall survival of the patients with the KIF20A-peptide vaccination was fairly improved (p = 0.0468, MST: 142 vs. 83 days). In comparison with the BSC patients (n = 81), overall survival of the vaccinated patients in Chiba Tokushukai Hospital was significantly improved (p = 0.0020, MST: 142 vs. 63 days) **(c)**.

The median overall survival time of 31 patients was 142 days, and the progression free survival period was 56 days (Figures 4a and 4b). In comparison with the control group without the vaccine treatment, who are the patients visited Chiba Tokushukai Hospital in the period between January 2007 and January 2009 (MST: 83 days), overall survival of the patients with the KIF20A-peptide vaccination was statistically significant (p = 0.0468, MST: 142 vs. 83 days) (Figure 4c). Moreover, MST of the patients who received BSC was 63 days. Compared to the control group in multi-center, Overall Survival of the vaccinated patients was significantly improved (p = 0.0020, MST: 142 vs. 63 days) (Figure 4c). Taken together, we concluded that the cancer vaccination utilizing KIF20A-derived peptide was significantly effective as immunotherapy against advanced pancreatic cancer.

### CTL response and injection site reactions

**Figure 5:**
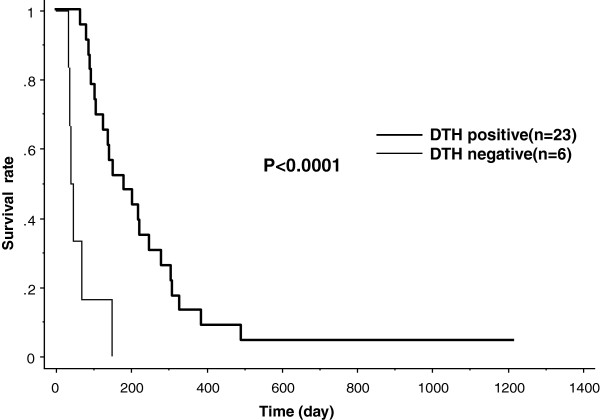
**Correlation between OS and ISR.** The local immune reactions at the site of injection were observed in 23 patients. MST of the patients who had injection site reaction was 182 days, while MST of the patients without such reaction (n = 6) was 42 days (p < 0.0001).

We expected that the number of CTL responded to KIF20A peptide may be associated with the efficacy of the vaccine treatment. Therefore, CTL response was measured by ELISPOT assay in 29 patients who received the vaccination at least one cycle (Table 2). Among them, CTL responses in 24 patients were comparable in pre- and post-vaccination. In 16 patients out of 23 (70%), the intensity of CTL response was increased (Table 2), determined by the algorithm flow chart [25]. Of note, strong CTL response specific to KIF20A-66 was observed two months after the start of the vaccination in the patient of case 9, who achieved CR. This response kept strong for one year, and it was detectable even 2 years after the drug was discontinued (Figure 2). A flow cytometry assay demonstrated that the number of KIF20A-66 specific TCR in CD8-positive T cells was consistent with the grades classified according to our algorithm flow chart [25] (Figure 3a), compared to the negative control stain utilizing HIV-dextramer (Figure 3b). Also, injection site reactions were observed in 23 patients. MST of the patients with positive skin reaction was 182 days, while that of the patients with negative reaction was 42 days (Figure 5). These results demonstrate that CTL response and ISRs could be employed as biological markers to rapidly diagnose the efficacy of the peptide vaccination. Consistent with these results, when the 29 patients were classified into two groups in regard to the content ratio of lymphocyte (more than 16% (n = 23) vs. less than 16% (n = 6)), the group with higher number of lymphocyte yielded better prognosis with statistical significance (p = 0.0296). This result suggests that the number of lymphocyte is positively associated with the survival of the patients.

## Discussion

Currently, there is no therapeutic strategy effective for the patients, whose pancreatic cancer is refractory to gemcitabine and TS-1. Combination therapy utilizing a couple of cytotoxic agents with gemcitabine has been investigated, but it has been failed to prove their clinical benefit so far [6-15]. We conducted an expression screening of proteins that were highly up-regulated in tumor cells, and not in normal cells, as a candidate of the target to develop novel anti-cancer drugs [20]. We successfully identified a member of kinesin super family protein 20A (KIF20A). Subsequently, we established an epitope peptide that were likely to be presented as an antigen in a HLA-A*2402- or HLA-A*0201-restricted manner [23,24,27]. In this report, we demonstrated that the KIF20A-derived peptide could improve the prognosis of the patients with advanced pancreatic cancer, suggesting that the KIF20A peptide vaccination is a promising approach as cancer immunotherapy.

In this clinical trial, we evaluated the safety and efficacy of KIF20A-66 peptide vaccine monotherapy for the patients with HLA-A*2402. This vaccine was well tolerated in the doses of 1.0 mg and 3.0 mg/body, although we do not exclude the possibility of two adverse events related to vaccination. The MST of 31 patients was 142 days in this phase I/II trial, indicating that vaccine treatment utilizing KIF20A-66 peptide provides survival benefit. Therefore, we concluded that the peptide vaccination improved overall survival period of the patients with advanced pancreatic cancer, who were resistant to chemotherapy. A placebo-controlled clinical trial should be required to further establish this peptide vaccine as a standard immunotherapy against pancreatic cancer.

We realized, during the course of peptide vaccination, that an induction of peptide-specific CTL and positive skin reaction were observed in the majority of the patients. We assure that these reactions could be employed as biomarkers of preferable clinical responses. Therefore, the number of CTL induced by peptide injection and the skin reaction at an injection site were analyzed. As we expected, high level of CTL response specific to KIF20A-66 peptide resulted in CR in case 9. The liver metastasis continuously shrunk even after the peptide vaccination was discontinued (Figure 1a), and there was no sign of recurrence or metastasis at the time of 40 months after the vaccination started. Since biopsy of the tumor lesion was not performed during or after the vaccination, there is no information regarding the tumor infiltrating lymphocyte (TIL). This example indicates that positive correlation between tumor shrinkage and immunological reactions is of clinically interest (Figure 2). On the other hand, there is no CTL induction detected in Case No. 4, 27, and 28, while objective shrinkages were observed in these patients during the course of treatment. Since the number of CTL is usually low in peripheral blood, the CTL induction is measured after the stimulation utilizing respective peptide and IL-2 to yield higher detection limit. Despite this procedure, it is assumed that the intensity of CTL induction and the efficacy of vaccine treatment are not necessarily correlated according to a linear function, possibly due to the high expression levels of MHC Class I and/or targeted antigen KIF20A in tumor cells. Therefore, development of sensitive and reliable methods to detect CTL is required to evaluate the results of peptide vaccine treatment in the patients.

The US FDA published the guidance for the therapeutic cancer vaccine [28], describing that it is hard to expect clinical benefit of the vaccine treatment for the patients after multiple chemotherapy regimens due to very poor immune status. However, unlike many trials tested so far utilizing other peptide vaccines, this clinical study was quite successful. Our results clearly demonstrate that therapeutic cancer vaccination is still a promising approach for advanced pancreatic cancer after the failure of standard chemotherapy. In general, patients with relapsed or recurrent metastatic disease receive multiple treatments for their cancer. These therapies may be detrimental to the immune system, and adequate time is required for the cancer vaccine to elicit a detectable immune response. Given such therapeutic conditions affect the results of peptide vaccination, the use of adjuvant setting and the cohort study during an early treatment of the vaccine may be necessary to better understand a cause-and-result relationship of cancer immunotherapy. Furthermore, it is important to develop the peptides with the higher immunogenicity against active oncoproteins. Indeed, we have examined several peptides derived from a variety of cancer-testis antigens that have the oncogenic activity, including KIF20A, DEPDC1, MPHOSPH1, URLC10(LY6K),TTK, KOC1(IMP3), CDCA1, RNF43, and TOMM34 [16,17,20,22-25,27,29]. We propose that the trial of the cocktail vaccine of these high immunogenic peptides including KIF20A-66 will provide with better treatment and cure for cancer.

## Abbreviations

HLA: Human leukocyte antigen; CR: Complete response; SD: Stable disease; PD: Progressive disease; MST: Median survival time; CTL: Cytotoxic T lymphocyte; 5-FU: 5-fluorouracil; ECOG: Eastern cooperative oncology group; RECIST: Response evaluation criteria in solid tumors; OS: Overall survival; PFS: Progression free survival; ISRs: Injection site reactions; IFA: Incomplete freund’s adjuvant; ELISPOT: Enzyme-linked immunospot; PBMC: Peripheral blood mononuclear cell; IFN: Interferon; CIC: Cancer immunotherapy consortium; SAE: Severe adverse event; PR: Partial response; TIL: Tumor infiltrating lymphocyte.

## Competing interests

The authors declare that they have no financial competing interest.

## Authors’ contribution

SA designed, performed, and evaluated clinical study. KT participated as the main coordinator and investigator regarding the immunological data analysis and evaluation. KY, HM, and HY analyzed control studies in their hospitals. SA wrote the manuscript. All authors read and approved the final manuscript.
